# Clinical characteristics of chronic liver disease with coronavirus disease 2019 (COVID-19): a cohort study in Wuhan, China

**DOI:** 10.18632/aging.103632

**Published:** 2020-08-28

**Authors:** Chaowei Li, Qingshi Chen, Jianwen Wang, Huasong Lin, Yalan Lin, Jinhuang Lin, Fangzhan Peng, Jiangmu Chen, Zhirong Yang

**Affiliations:** 1 Department of Gastroenterology, The Second Affiliated Hospital of Fujian Medical University, Quanzhou, China; 2 Department of Endocrinology, The Second Affiliated Hospital of Fujian Medical University, Quanzhou, China; 3 Department of Pulmonary Diseases and Tuberculosis, Jin Yin-Tan Hospital, Wuhan, China; 4 Department of Neurology, The Second Affiliated Hospital of Fujian Medical University, Quanzhou, China; 5 Department of Respiratory and Critical Care Medicine, The Second Affiliated Hospital of Fujian Medical University, Quanzhou, China; 6 Department of Neurology, The Affiliated Southeast Hospital of Xiamen University, Zhangzhou, China; 7 Department of Emergency Management, The Second Affiliated Hospital of Fujian Medical University, Quanzhou, China; 8 Department of Surgical, The Fujian Pinghe County People's Hospital, Zhangzhou, China

**Keywords:** COVID-19, LOS, severity, NLR, mortality

## Abstract

Background: Previous work has described acute liver injury (ALI) in coronavirus disease 2019 (COVID-19) pneumonia patients, However, there is limited analyses available investigating chronic liver disease (CLD) in COVID-19 patients. This study aimed to investigate clinical characteristics and outcomes of CLD confirmed in COVID-19 patients.

Results: A total of 104 cases (each group containing 52 patients) were analyzed in this study. The CLD group showed an average of 14 (10.0~21.2) length of stay (LOS) days, compared to the group without CLD that only showed an average of 12.5 (10~16) LOS days (Relative Risk [RR] = 1.34, 95% CI (1.22~1.48), P<0.001; Adjusted Relative Risk was 1.24 (95% CI: 1.12~1.39)). The CLD group contained a higher mortality rate and slight liver injury. Furthermore, COX regression model analyses suggested that the neutrophil-to-lymphocyte ratio (NLR) was an independent predictor of mortality risk (P < 0.001) in the CLD group. Additionally, a high NLR significantly correlated with a shorter overall survival (P <0.001).

Conclusions: COVID-19 patients also diagnosed with CLD suffered longer LOS, slight liver injuries and a higher mortality when compared to COVID-19 patients without CLD. The NLR was an independent risk factor for in-hospital deaths. Increased expression of NLR was an indicator of poor prognosis in COVID-19 patients with CLD. Thus, COVID-19 patients diagnosed with CLD and who show a higher NLR need additional care.

Methods: A retrospective cohort study was performed at the Wuhan Jin Yin-tan Hospital from February 2, 2020 to April 2, 2020. COVID-19 patients diagnosed with CLD or not diagnosed with CLD were enrolled in this study. The clinical characteristics and outcomes of these patients were compared.

## INTRODUCTION

Coronavirus disease 2019 (COVID-19) pneumonia, emerged in Wuhan (Hubei, China), has rapidly spread worldwide, infecting over 3.48 million patients. Previous work has mainly described acute liver injury (ALI) in general COVID-19 pneumonia patients [1–4]. There is little research available that focuses on patients with liver disease, especially chronic liver disease (CLD). Although some studies and reviews had reported that CLD is not associated with severity or mortality of COVID-19, all these used small sample sizes and most likely failed to analyze the characteristics and mortality rates of these patients. To our knowledge, the study presented here is the first to investigate clinical features and outcomes of CLD patients who have been diagnosed with COVID-19 pneumonia in a largest cohort study.

## RESULTS

**Figure 1:**
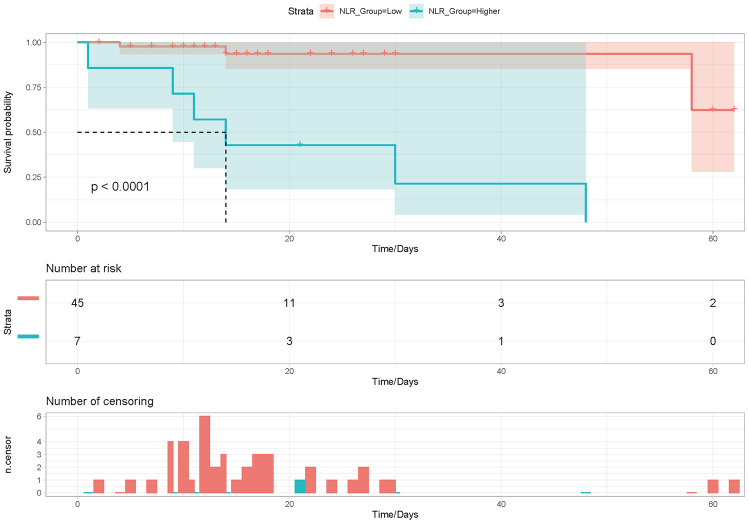
**Kaplan-Meier analysis of overall survival (OS) according to NLR expression in CLD with COVID-19 infection patients.**

A total 104 patients were analyzed in this study, with both groups containing 52 patients (Table 1). In addition to CLD, a total of 39 (37.5%) patients showed other comorbidities. The median age of the patients analyzed was 59 (SD 12.9) years of age and a total of 39 (37.5%) patients were female. The most common symptoms experienced by the patients included cough (85[81.7%]), expectoration (38[36.5%]), dyspnea (19[18.3%]), and fatigue or myalgia (13 [12.5%]). All patients showed bilateral infiltrates on chest CT, while 92 (88.5%) patients had bilateral infiltrates. A total of 34.6%,35.6% and 5.77% of patients showed elevated ALT, AST and TBil levels, respectively. There was a total of 9 death patients in the CLD group, including 6 patients died of respiratory and circulatory failure, 3 patients died of multiple organ dysfunction syndrome (MODS). There were no significant differences observed in demographics, initial common symptoms, laboratory findings without lymphocyte count, PLT, INR, Glu IL-6 or PCT levels, liver function and treatment when comparing the two groups (P > 0.05; Table 1). The CLD group had showed a LOS of 14 (10.0~21.2) days compared to12.5 (10~16) for the non- CLD group (Relative Risk [RR] = 1.34, 95% CI (1.22~1.48), P<0.001; Adjusted RR was 1.24(95% CI: 1.12–1.39)) (Table 2). However, no differences in severity outcome were observed between the two groups ((Hazard Ratio [HR] = 1.78, 95% CI (0.80~4.04), P=0.16; Adjusted HR was 1.19, 95% CI(0 .45~3.19), P=0.73) (Table 2). There was no difference in severity ratio between the two groups (39 [37.5%] vs 16 [30.8%], p=0.22). The CLD group showed a higher mortality rate (9 [8.7%] vs 0[0.0%]) and slight liver injuries compared to the non-CLD group. Furthermore, univariate and multivariate COX regression analyses were performed to explore risk factors for death in the CLD group. Univariate survival analyses revealed that age, NLR, GLU and PCT were risk factors for death. However, only the NLR (OR, 1.04; 95% CI, 1.01–1.06) was found to be an independent predictor of death based on the multivariate analysis. (Table 3). To further assess prognostic significance of NLR in CLD patients with COVID-19, Kaplan-Meier survival analysis was performed to analyze overall survival (OS) (cutoff = 4.00). CLD patients with high NLR showed a significantly shorter OS (Figure 1, P <0.001). Thus, CLD was not associated with severity, but was associated with LOS, liver injury and mortality in patients diagnosed with COVID-19. Furthermore, NLR was shown to be an independent risk factor and prognostic factor for mortality in CLD patients confirmed to have COVID-19.

**Table 1:** Demographics and clinical features of patients with COVID-19.

	**All patients (N= 104)**	**Non-CLD (n = 52)**	**CLD (n = 52)**	***P***
Demographics				
Age	59 ± 12.9	59.7 ± 14	58.2 ± 11.7	0.58
sex, n (%)				
Female	39 (37.5)	19 (36.5)	20 (38.5)	1
Male	65 (62.5)	33 (63.5)	32 (61.5)	
Comorbidities, n (%)				
No	65 (62.5)	33 (63.5)	32 (61.5)	1
Yes	39 (37.5)	19 (36.5)	20 (38.5)	
Smoking, n (%)				
No	40 (38.5)	20 (38.5)	20 (38.5)	1
Yes	64 (61.5)	32 (61.5)	32 (61.5)	
Initial common symptoms				
Dyspnoea, n (%)				0.13
No	85 (81.7)	46 (88.5)	39 (75)	
Yes	19 (18.3)	6 (11.5)	13 (25)	
Cough, n (%)				
No	19 (18.3)	9 (17.3)	10 (19.2)	1
Yes	85 (81.7)	43 (82.7)	42 (80.8)	
Expectoration, n (%)				
No	66 (63.5)	28 (53.8)	38 (73.1)	0.07
Yes	38 (36.5)	24 (46.2)	14 (26.9)	
Myalgia or fatigue, n (%)				
No	91 (87.5)	46 (88.5)	45 (86.5)	1
Yes	13 (12.5)	6 (11.5)	7 (13.5)	
Sore throat, n (%)				
No	104 (100)	52 (100)	52 (100)	1
Thoracodynia, n (%)				
No	104 (100)	52 (100)	52 (100)	1
Systolic Pressure	125(117.8,133.3)	125 (118.0, 130.5)	125.5(116.3, 135.5)	0.39
Respiratory Rate	22 (20, 24)	22 (20, 23)	22 (20, 25)	0.51
Laboratory findings				
White blood cell count (×10^9^ cells per L)	5.5 (4.4, 7.36)	5.5 (4.36, 6.86)	5.48 (4.58, 8.02)	0.22
Neutrophil count (×10^9^ cells per L)	3.85(2.96, 5.39)	3.79 (2.96, 5.06)	3.92(2.91, 5.85)	0.69
Lymphocyte count (×10^9^ cells per L)	0.88(0.69, 1.18)	1.02 (0.85, 1.24)	0.79(0.55, 1.04)	< 0.001
MNM (×10^9^ cells per L)	0.4 ± 0.2	0.4 ± 0.2	0.4 ± 0.2	0.39
Hb (g/L)	124(114.0,134.3)	124(114.3, 132.0)	124(114.0,136.3)	0.49
PLT (×10^9^ per L)	211.5(164, 268)	233.5(183.75, 296)	186(155, 230)	<0.001
INR	0.96(0.9, 1.04)	0.94 (0.9, 0.99)	1 (0.92, 1.12)	0.03
Potassium (mmol/L)	4.1 ± 0.6	4 ± 0.6	4.2 ± 0.6	0.18
Sodium (mmol/L)	140.5 (139, 142)	141 (139, 143)	140 (138, 142)	0.13
Cl (mmol/L)	106 (104, 108)	107 (105, 108)	106 (103, 108)	0.08
BUN	4 (3.4, 5.23)	4 (3.3, 5.05)	4.1 (3.5, 5.7)	0.28
Cr (μmol/L)	70 (59.45, 79.98)	70.65(59.45, 82.05)	69.85(59.52, 78.15)	0.69
Glu (mmol/L)	5.8 (5.07, 7.05)	5.65 (5, 6.2)	6.3 (5.27, 7.7)	0.02
CK (U/L)	65(42.75, 185.25)	63 (41.00, 143.25)	73 (47.50, 208.25)	0.23
IL-6 (mmol/L)	8.52(6.52,11.52)	7.77 (6.5, 10.52)	9.58 (7.15, 15.45)	0.04
Infection, n (%)				
No	29 (27.9)	15 (28.8)	14 (26.9)	1
Yes	75 (72.1)	37 (71.2)	38 (73.1)	
PCT, Median (IQR)	0 (0, 0.07)	0 (0, 0.05)	0.05 (0, 0.23)	< 0.001
Chest CT				
Lobi Pulmonis, n (%)				
Unilateral	12 (11.5)	5 (9.6)	7 (13.5)	0.76
Bilateral	92 (88.5)	47 (90.4)	45 (86.5)	
Ground-glass opacity, n (%)				
No	45 (43.3)	23 (44.2)	22 (42.3)	1
Yes	59 (56.7)	29 (55.8)	30 (57.7)	
ALT (IU/L, baseline)	36.5(22.75,63.25)	42.5 (22.75, 68)	36 (24.25, 57.5)	0.62
AST (IU/L, baseline)	33 (25, 50)	32 (25, 46.5)	36.5 (25.75, 51.5)	0.4
Total bilirubin(μmol/L)	12.85 (10.38, 16.22)	11.95 (10.2, 14.12)	13.3 (10.95, 17.5)	0.1
ALB(g/L)	32 ± 4.2	31.3 ± 3.5	32.7 ± 4.7	0.08
PT(s)	11.25 (10.5, 12)	11 (10.5, 11.7)	11.65 (10.57, 12)	0.05
PTA	108.35 (89, 127.55)	111 (92.78, 130.6)	105.55 (88.4,124.35)	0.52
Treatment				
Antibiotic therapy, n (%)				
No	18 (17.3)	7 (13.5)	11 (21.2)	0.44
Yes	86 (82.7)	45 (86.5)	41 (78.8)	
Use of corticosteroid, n (%)				
No	79 (76)	43 (82.7)	36 (69.2)	0.17
Yes	25 (24)	9 (17.3)	16 (30.8)	
Oxygen support, n (%)				
No	19 (18.3)	7 (13.5)	12 (23.1)	0.31
Yes	85 (81.7)	45 (86.5)	40 (76.9)
Ventilation, n (%)				
Non-invasive ventilation	7 (6.7)	2 (3.8)	5 (9.6)	0.1
Invasive mechanical ventilation	6 (5.8)	1 (1.9)	5 (9.6)	
NO ventilation	91 (87.5)	49 (94.2)	42 (80.8)	
Prone position ventilation, n (%)				
No	103 (99)	52 (100)	51 (98.1)	1
Yes	1 (1)	0 (0)	1 (1.9)	
ECMO, n (%)				
No	104 (100)	52 (100)	52 (100)	1
Nebulization inhalation, n (%)				
No	99 (95.2)	50 (96.2)	49 (94.2)	1
Yes	5 (4.8)	2 (3.8)	3 (5.8)	
Vasoconstrictor, n (%)				
No	103 (99)	52 (100)	51 (98.1)	1
Yes	1 (1)	0 (0)	1 (1.9)	
Immunoglobulin therapy, n (%)				
No	86 (82.7)	47 (90.4)	39 (75)	0.07
Yes	18 (17.3)	5 (9.6)	13 (25)	

Abbreviations: CLD, Chronic liver disease; ALP, alkaline phosphatase; ALT, alanine transaminase; AST, aspartate; INR, International Normalized Ratio; PT, Prothrombin time; *PLT*, *blood* *platelet;* *CK,* *creatine kinase;* BUN, blood urea nitrogen; *RR*: Respiratory Rate; MNM: monocyte counts; IL-6, interleukin-6; ECMO, extracorporeal membrane oxygenation.

**Table 2:** Unadjusted and adjusted risk ratios of LOS, severity, and mortality in COVID-19.

**Outcome**	**β**	**Unadjusted risk ratio**	**95% CI**	***P***	**β**	**Adjusted risk ratio**	**95% CI**	***P***
LOS Outcome								
	0.30	1.34	(1.22~1.48)	<0.01	0.22	1.24	1.12~1.39	<0.001
Severity Outcome								
	0.58	1.78	0.80~4.04	0.16	0.17	1.19	0.45~3.19	0.73
Mortality Rate Outcome								
		CLD (n = 52)				Non-CLD (n = 52)		*P*
	Survivors	43 (82.7)				52 (100)		<0.01
	Death	9 (17.3)				0 (0)		
Liver Injury Outcome								
	Total (N = 104)		CLD (n = 52)			Non-CLD (n = 52)		*P*
ALT	37 (24.75, 57.25)		39.5 (28.75, 55.5)			33.5 (23, 62)		0.47
AST	28.5 (19, 38.75)		30 (23, 49.5)			24 (17.75, 34.25)		< 0.001
TBil	10.05 (6.7, 15.12)		13.9 (7.18, 20.8)			8.6 (6.7, 11.93)		< 0.001

(CLD group verse Non-CLD group).

**Table 3:** Unadjusted and adjusted risk factors of mortality for CLD group.

	**β**	**Unadjusted hazard ratio**	**95%CI**	***P***	**β**	**Adjusted hazard ratio**	**95%CI**	***P***
Age	2.13493	8.46	1.73~41.37	0.0084				
NLR	0.046	1.05	1.02~1.07	<0.001	0.03624	1.04	1.01~1.06	<0.001
GLU	0.24	1.27	1.03~1.57	0.028				
IL-6	0.005009	1.01	0.97 ~1.05	0.799				
PCT	2.28	9.74	1.85~51.20	<0.001				
INR	0.04832	0.95	0.45~2.02	0.9				

NLR, neutrophil-to-lymphocyte ratio.

## DISCUSSION

COVID-19 patients diagnosed with CLD showed a prolong LOS, slightly liver injuries and higher mortality rates compared to general COVID-19 patients. Furthermore, the NLR was found to be an independent risk factor for mortality in COVID-19 patients with CLD. Moreover, increased expression of NLR is an independent indicator of poor prognosis in COVID-19 patients diagnosed with CLD.

Previous work conducted by our team and others has shown that COVID-19 patients were more prone to liver injury whether general patients or critically ill patients [1, 5–11]. In the other hand, a severe outcome of COVID-19 disease was associated with liver dysfunction [12]. Although there are some reports that liver injury is uncommon in these cases [13], CLD should also be analyzed related to COVID-19 risk due to poor immune function. However, for CLD patients, some studies [5] showed that no patient should be receiving ICU care. Similarly, there were no significant differences for AST and ALT between the ICU and non-ICU groups, where both groups showed a normal distribution [5]. There were also no differences in liver function between survivors and non-survivors [14]. At the same time, other studies revealed that COVID-19 patients diagnosed with CLD did not show associations with severity or mortality [6]. This research only included rare CLD patients, where in some studies only a single CLD patient was included. Thus, these studies failed to explore the risk of severity or mortality associated with CLD. Here, we focused on all CLD confirmed COVID-19 cases presented in the Jin Yin-tan Hospital. CLD patients showed a prolonged LOS, slight liver injures and an increased risk for death, but not severity. To the best of our knowledge, this is the first study focusing on COVID-19 patients diagnosed with CLD. The COVID-19 with CLD group showed an increased lymphocyte count as well as increased IL-6 and PCT levels, suggesting pathogenic effects from excessive inflammation in acute lung injury caused by COVID-19 infection. Inflammation may reflect disease severity and defects in innate immune regulation, especially in CLD patients who have poor immune function [13]. At the same time, blood sugar levels were elevated to support the specific energetic demands needed by inflammatory responses [15, 16]. This work also revealed that the CLD group had relatively low PLT and increased total bilirubin and INR levels, supporting hepatic dysfunction in the activation of coagulative and fibrinolytic, which consistent with previous studies [13, 17] In this study, risk of CLD was found to be related to LOS in COVID-19. Compared to patients without CLD, the patient group diagnosed with COVID-19 and CLD showed a prolonged LOS. With a prolonged LOS, CLD may improve the risk of nosocomial infections (NIs), which may result in a worse prognosis. Similarly, the CLD group showed an increased mortality rate and higher incidence of liver injury. Interestingly, although the risk of CLD was positively associated with LOS, liver function risk and mortality, it was negatively associated to severity which was consistent with other studies [6, 13]. We performed additional work to explore risk factors related to mortality in the CLD group. The most important finding was that the NLR was associated with mortality and severity, suggesting it as a potential indicator for poor prognosis in CLD patients diagnosed with COVID-19 infection. The high NLR accounts for increased neutrophil and decreased lymphocyte counts, reflecting systemic inflammation. Systemic inflammation is believed to play an important role in the severity of virus-induced disease, such as COVID-19. Meanwhile, patients diagnosed with CLD have poor immune function. Thus, CLD may aggravate the dysregulation of the immune response associated with COVID-19 [18]. An easily accessible and less costly biological marker for systemic inflammatory diseases, NLR has been reported as an independent risk factor for the severity and mortality in COVID-19 [3, 18, 19], especially in elder or male patients [19]. Since NLR could be quickly calculated based on a blood routine test on admission, clinicians may identify high risk COVID-19 patients at an early stage. Thus, treatments can be modified accordingly to reduce the in-hospital death. In particular, this study confirmed the risk of NLR associated with mortality in CLD patients.

This study also exhibits some limitations. (a) The sample size was limited to a single-center hospital clinical characteristics COVID-19 patients diagnosed with CLD should be verified with a randomized controlled trial enrolling more patients. (b) The diagnosis of CLD needs systematic testing such as a biopsy of the liver, blood tests and imaging including ultrasound. This study was unable to widely diagnose all CLD cases through systematic testing when the COVID-19 outbreak occurred in Wu Han since there were limited medical resources. However, CLD was diagnosed based on written medical and *oral health records with the goal to minimize bias* of diagnosis. (c) The study population included older COVID-19 patients diagnosed with CLD, suggesting that these conclusions may not be applicable to younger patients. In the future, a larger multicenter study analyzed CLD patients diagnosed with COVID-19 is needed to further understand the pathological mechanisms behind CLD associated with COVID-19. This would contribute to further knowledge defining the clinical characteristics and outcome for these patients.

## CONCLUSIONS

This retrospective study revealed that COVID-19 patients diagnosed with CLD showed a longer LOS, slight liver injuries and higher mortality compared to general COVID-19 patients. The NLR was found to be an independent risk factor for in-hospital deaths. Increased expression of NLR was found to be a potential indicator for poor prognosis in COVID-19 patients diagnosed with CLD. Thus, CLD patients with COVID-19 who have a higher NLR should be critically cared for.

## MATERIALS AND METHODS

### Study design, participants and data collection

The retrospective cohort study presented here included all CLD and random non-CLD patients at Wuhan Jin Yin-tan Hospital. Wuhan Jin Yin-tan Hospital (Wuhan Isolation Hospital) is known to have treated the largest number of COVID-19 patients. All enrolled patients were treated from February 2, 2020 to April 2, 2020. The diagnostic and treatment criteria of COVID-19 and its severity were based on guidelines provided by the WHO and China Trial Seventh Edition. Patients diagnosed with acute liver injury or who showed incomplete medical records were excluded from this study. Clinical data such as demographics, initial symptoms, laboratory findings, chest CT pneumonia compromise and treatment was reviewed using digital medical records by the Fujian Medical Team to aid Wuhan Jin Yin-tan Hospital. Patients were divided into two groups including the COVID-19 with CLD group and non-CLD group. CLD was defined as a progressive deterioration of liver functions, leading to fibrosis and cirrhosis of liver parenchyma. It refers to liver disease at least 6 months. CLD consists of diverse liver pathologies including hepatocellular carcinoma, liver cirrhosis, and inflammation (chronic hepatitis). Our team diagnosed CLD based on clinical features. The COVID-19 with CLD group included all CLD patients that were diagnosed with chronic viral hepatitis B and C, autoimmune liver disease, cryptogenic liver cirrhosis, NAFLD, methotrexate related liver fibrosis and alcoholic liver disease. At the same time, we used computer-generated random same size to enroll non-CLD group during the same period. We analyzed the clinical characteristics of all patients, then compared the baseline information and the outcome of LOS, severity, mortality rate and liver function. This retrospective cohort study approved by Ethics Commission of Jin Yin-tan hospital, Wuhan (KY-2020–55.01).

### Statistical analysis

The mean ± SD or median (IQR) value and number (%) were used to descriptive data of continuous and categorical variables. For continuous variables, independent group t tests were used to compare the two group or Mann–Whitney test was performed when data were normally distributed. For categorical variables, the χ ^2^ or Fisher exact tests were performed to compared the two groups. Furthermore, Poisson regression was used to verify independent risk of CLD for LOS. Stepwise Logistic Regression models were used to test independent risks of CLD for severity. Cox models were used to calculate the hazard ratio of mortality in the CLD group. Kaplan-Meier survival analysis was used to analyze overall survival (OS) of the CLD group based on neutrophil-to-lymphocyte ratio (NLR) levels. R project (version 3.6.0) was used to perform all statistical analyses. Statistical significance was recognized at a P value of 0.05 or less.

## References

[1] Xie H, Zhao J, Lian N, Lin S, Xie Q, Zhuo H. Clinical characteristics of non-ICU hospitalized patients with coronavirus disease 2019 and liver injury: a retrospective study. Liver Int. 2020; 40:1321–26. 10.1111/liv.14449 32239591 PMC7228333

[2] Xu L, Liu J, Lu M, Yang D, Zheng X. Liver injury during highly pathogenic human coronavirus infections. Liver Int. 2020; 40:998–1004. 10.1111/liv.14435 32170806 PMC7228361

[3] Zhang Y, Zheng L, Liu L, Zhao M, Xiao J, Zhao Q. Liver impairment in COVID-19 patients: a retrospective analysis of 115 cases from a single centre in Wuhan city, China. Liver Int. 2020. [Epub ahead of print]. 10.1111/liv.14455 32239796

[4] Zhao D, Yao F, Wang L, Zheng L, Gao Y, Ye J, Guo F, Zhao H, Gao R. A comparative study on the clinical features of COVID-19 pneumonia to other pneumonias. Clin Infect Dis. 2020. [Epub ahead of print]. 10.1093/cid/ciaa247 32161968 PMC7108162

[5] Huang C, Wang Y, Li X, Ren L, Zhao J, Hu Y, Zhang L, Fan G, Xu J, Gu X, Cheng Z, Yu T, Xia J, et al. Clinical features of patients infected with 2019 novel coronavirus in Wuhan, China. Lancet. 2020; 395:497–506. 10.1016/S0140-6736(20)30183-5 31986264 PMC7159299

[6] Lippi G, de Oliveira MH, Henry BM. Chronic liver disease is not associated with severity or mortality in coronavirus disease 2019 (COVID-19): a pooled analysis. Eur J Gastroenterol Hepatol. 2020. [Epub ahead of print]. 10.1097/MEG.0000000000001742 32282549 PMC7690326

[7] Qin C, Zhou L, Hu Z, Zhang S, Yang S, Tao Y, Xie C, Ma K, Shang K, Wang W, Tian DS. Dysregulation of immune response in patients with COVID-19 in Wuhan, China. Clin Infect Dis. 2020. [Epub ahead of print]. 10.1093/cid/ciaa248 32161940 PMC7108125

[8] Ruan Q, Yang K, Wang W, Jiang L, Song J. Correction to: clinical predictors of mortality due to COVID-19 based on an analysis of data of 150 patients from Wuhan, China. Intensive Care Med. 2020; 46:1294–97. 10.1007/s00134-020-06028-z 32253449 PMC7131986

[9] Ji D, Zhang D, Xu J, Chen Z, Yang T, Zhao P, Chen G, Cheng G, Wang Y, Bi J, Tan L, Lau G, Qin E. Prediction for progression risk in patients with COVID-19 pneumonia: the CALL score. Clin Infect Dis. 2020. [Epub ahead of print]. 10.1093/cid/ciaa414 32271369 PMC7184473

[10] Yang X, Yu Y, Xu J, Shu H, Xia J, Liu H, Wu Y, Zhang L, Yu Z, Fang M, Yu T, Wang Y, Pan S, et al. Clinical course and outcomes of critically ill patients with SARS-CoV-2 pneumonia in Wuhan, China: a single-centered, retrospective, observational study. Lancet Respir Med. 2020; 8:475–81. 10.1016/S2213-2600(20)30079-5 32105632 PMC7102538

[11] Chen R, Liang W, Jiang M, Guan W, Zhan C, Wang T, Tang C, Sang L, Liu J, Ni Z, Hu Y, Liu L, Shan H, et al, and Medical Treatment Expert Group for COVID-19. Risk factors of fatal outcome in hospitalized subjects with coronavirus disease 2019 from a nationwide analysis in China. Chest. 2020; 158:97–105. 10.1016/j.chest.2020.04.010 32304772 PMC7158802

[12] Youssef M, Hussein M, Attia AS, Elshazli R, Omar M, Zora G, Farhoud A, Elnahla A, Shihabi A, Toraih E, Fawzy M, Kandil E. COVID-19 and liver dysfunction: a systematic review and meta-analysis of retrospective studies. J Med Virol. 2020; 10:1002. 10.1002/jmv.26055 32445489 PMC7283797

[13] Bangash MN, Patel J, Parekh D. COVID-19 and the liver: little cause for concern. Lancet Gastroenterol Hepatol. 2020; 5:529–30. 10.1016/S2468-1253(20)30084-4 32203680 PMC7270582

[14] Huang J, Cheng A, Kumar R, Fang Y, Chen G, Zhu Y, Lin S. Hypoalbuminemia predicts the outcome of COVID-19 independent of age and co-morbidity. J Med Virol. 2020; 10:1002. 10.1002/jmv.26003 32406952 PMC7273060

[15] Brand A, Singer K, Koehl GE, Kolitzus M, Schoenhammer G, Thiel A, Matos C, Bruss C, Klobuch S, Peter K, Kastenberger M, Bogdan C, Schleicher U, et al. LDHA-associated lactic acid production blunts tumor immunosurveillance by T and NK cells. Cell Metab. 2016; 24:657–71. 10.1016/j.cmet.2016.08.011 27641098

[16] Pearce EL, Poffenberger MC, Chang CH, Jones RG. Fueling immunity: insights into metabolism and lymphocyte function. Science. 2013; 342:1242454. 10.1126/science.1242454 24115444 PMC4486656

[17] Wang D, Hu B, Hu C, Zhu F, Liu X, Zhang J, Wang B, Xiang H, Cheng Z, Xiong Y, Zhao Y, Li Y, Wang X, Peng Z. Clinical characteristics of 138 hospitalized patients with 2019 novel coronavirus-infected pneumonia in Wuhan, China. JAMA. 2020; 323:1061–69. 10.1001/jama.2020.1585 32031570 PMC7042881

[18] Liu Y, Du X, Chen J, Jin Y, Peng L, Wang HH, Luo M, Chen L, Zhao Y. Neutrophil-to-lymphocyte ratio as an independent risk factor for mortality in hospitalized patients with COVID-19. J Infect. 2020; 81:e6–12. 10.1016/j.jinf.2020.04.002 32283162 PMC7195072

[19] Liu J, Liu Y, Xiang P, Pu L, Xiong H, Li C, Zhang M, Tan J, Xu Y, Song R, Song M, Wang L, Zhang W, et al. Neutrophil-to-lymphocyte ratio predicts critical illness patients with 2019 coronavirus disease in the early stage. J Transl Med. 2020; 18:206. 10.1186/s12967-020-02374-0 32434518 PMC7237880

